# A Modifier Screen for Bazooka/PAR-3 Interacting Genes in the *Drosophila* Embryo Epithelium

**DOI:** 10.1371/journal.pone.0009938

**Published:** 2010-04-01

**Authors:** Wei Shao, Johnny Wu, Jeyla Chen, Donghoon M. Lee, Alisa Tishkina, Tony J. C. Harris

**Affiliations:** Department of Cell and Systems Biology, University of Toronto, Toronto, Ontario, Canada; Institut Pasteur, France

## Abstract

**Background:**

The development and homeostasis of multicellular organisms depends on sheets of epithelial cells. Bazooka (Baz; PAR-3) localizes to the apical circumference of epithelial cells and is a key hub in the protein interaction network regulating epithelial structure. We sought to identify additional proteins that function with Baz to regulate epithelial structure in the *Drosophila* embryo.

**Methodology/Principal Findings:**

The *baz* zygotic mutant cuticle phenotype could be dominantly enhanced by loss of known interaction partners. To identify additional enhancers, we screened molecularly defined chromosome 2 and 3 deficiencies. 37 deficiencies acted as strong dominant enhancers. Using deficiency mapping, bioinformatics, and available single gene mutations, we identified 17 interacting genes encoding known and predicted polarity, cytoskeletal, transmembrane, trafficking and signaling proteins. For each gene, their loss of function enhanced adherens junction defects in zygotic *baz* mutants during early embryogenesis. To further evaluate involvement in epithelial polarity, we generated GFP fusion proteins for 15 of the genes which had not been found to localize to the apical domain previously. We found that GFP fusion proteins for *Drosophila* ASAP, Arf79F, CG11210, Septin 5 and Sds22 could be recruited to the apical circumference of epithelial cells. Nine of the other proteins showed various intracellular distributions, and one was not detected.

**Conclusions/Significance:**

Our enhancer screen identified 17 genes that function with Baz to regulate epithelial structure in the *Drosophila* embryo. Our secondary localization screen indicated that some of the proteins may affect epithelial cell polarity by acting at the apical cell cortex while others may act through intracellular processes. For 13 of the 17 genes, this is the first report of a link to *baz* or the regulation of epithelial structure.

## Introduction

Epithelial structure is essential for the development and homeostasis of multicellular organisms (for reviews see [1]–[9]). Each cell in an epithelial sheet has an apical domain facing the sheet surface and a basolateral domain facing underlying tissue. This polarity is tightly linked to epithelial structure. Adherens junctions (AJs; formed from cadherin adhesion molecules and the β-catenin (Armadillo; Arm) and α-catenin adaptor proteins) form around the circumference of the apical domain and connect neighbouring cells. Actin associates with AJs but also localizes laterally and basally. Similarly, microtubules (MTs) are organized in specific apical, lateral and basal networks, while intracellular trafficking pathways direct specific cargo to the apical or basolateral domains. This polarized organization of epithelial cells controls transport between body compartments, and is critical for the development and maintenance of epithelial structure.

Studies from *C. elegans*, *Drosophila* and mammalian systems have revealed specific polarity complexes that regulate polarized epithelial structure (for reviews see [1], [10]–[18]). The Baz (fly PAR-3) complex (cytoplasmic Baz, PAR-6, aPKC and Cdc42) and the Crumbs (Crb) complex (transmembrane Crb, and cytoplasmic Stardust and Patj) are apical cues, whereas the Discs large (Dlg) complex (cytoplasmic Dlg, Lethal giant larvae and Scribble) is a basolateral cue. Mutations disrupting these polarity complexes lead to epithelial breakdown and depolarization, and interactions between the complexes form key elements of the polarity establishment hierarchy. Certain interactions recruit and maintain proteins in the apical domain. Baz and PAR-6 recruit Crb and Patj, respectively [19], [20]; and aPKC stabilizes apical Crb [21]. In turn, Crb stabilizes AJs and Baz [22]–[24]. Other interactions help segregate the apical and basolateral domains. Crb has a mutually antagonistic relationship with the basolateral Dlg complex [19], [25]. Apical aPKC activity can exclude both Lgl [20] and the basolateral kinase PAR-1 [26], while Lgl and PAR-1 in the basolateral domain displace aPKC and Baz, respectively [20], [27].

Baz/PAR-3 is a molecular scaffold with no predicted enzyme activity. It is a cytoplasmic protein with three main regions, an N-terminal region, a central region with three PDZ protein interaction domains, and a C-terminal region. The N-terminal region can homo-oligomerize [28]. PAR-6 binds the first PDZ domain of PAR-3 and aPKC interacts with the C-terminal region of PAR-3 [15]. However, studies in *C. elegans* and *Drosophila* indicate that Baz/PAR-3 can also function separately from PAR-6 and aPKC [29], [30]. Studies from a variety of cell types have shown that Baz and/or PAR-3 can also directly interact with components of cell adhesion complexes; Arm and Echinoid (Ed) [31] and p75NTR [32]; actin cytoskeleton regulators; Tiam 1 [33], PTEN [34], LIM kinase 2 [35], and Rho-kinase [36]; the microtubule motors KIF3A [37], [38] and dynein [39]; Numb [40]; the ubiquitin ligase Smurf2 [41], and lipids [42].

*Drosophila* embryogenesis provides an excellent model to study how Baz/PAR-3 regulates epithelial structure. In *Drosophila*, Baz plays a key role in positioning AJs as the first epithelium forms [24], [43]. This involves an unknown actin-based apical scaffold and the positioning of Baz in proximity to apical MT minus-ends by dynein [29]. Baz also positions an actin-regulator (Bitesize) that affects AJs just after they have formed [44]. Moreover, Baz becomes planar polarized in these later tissues suggesting a role in polarized junctional modeling [45]. Other work has shown roles for Baz in regulating the endocytosis and recycling of AJs [46], [47] and apical proteins [48]. This diverse set of cellular activities suggests that Baz interacts directly or indirectly with a variety of epithelial polarity regulators.

We performed a genetic screen to identify additional players that function with Baz to regulate epithelial structure in the *Drosophila* embryo. At the end of embryogenesis, the epidermis secretes a protective cuticle which provides an assay for detecting defects in epidermal structure and patterning [49]. Maternal/zygotic *baz* mutants display a severe cuticle phenotype with only scattered scraps of cuticle produced by residual epithelia [19]. However, zygotic *baz* mutants have a maternal supply of *baz* gene product that can produce a largely intact cuticle with only one or two holes [25], [50]. The activity of this maternal supply can be reduced by reducing levels of proteins that function with Baz. For example, reduction of dynein heavy chain levels dominantly enhances the zygotic *baz* mutant cuticle phenotype [29].

In a pilot screen, we found that the *baz* zygotic mutant cuticle phenotype could be dominantly enhanced to varying degrees by reducing the levels of known polarity regulators. To identify new polarity regulators we screened molecularly defined deficiencies of chromosomes 2 and 3. 37 of the deficiencies showed strong dominant enhancement of the *baz* zygotic mutant cuticle phenotype. We used deficiency mapping, bioinformatics, and available single gene mutations to identify 17 interacting genes. Immunofluorescence microscopy showed that loss of function of these genes enhanced AJ defects in zygotic *baz* mutants in early embryonic epithelia. Surprisingly, the individual cuticle phenotypes for the interacting alleles were relatively mild. Nonetheless, seven of the 17 proteins localize to the apical cortex. Some of the identified genes are known polarity regulators, but most have not been previously linked to *baz* or the regulation of epithelial structure.

## Methods

### Fly stocks and genetics

Descriptions of genetic mutations and constructs can be found on FlyBase (http://flybase.bio.indiana.edu). *baz^Xi106^*, *par1^w3^*, *apkc^K06403^*, *shg^R69^*, *ed^kg^*, *hk* ^11^ and *asp^L18^* mutants were gifts of A. Wodarz (Göttingen Univ., Germany), H. McNeill (Univ. of Toronto, Canada), C. Doe (Univ. of Oregon, USA), U. Tepass (Univ. of Toronto, Canada), M. Peifer (Univ. of North Carolina, USA), H. Kramer (Univ. of Texas Southwestern, USA) and D. Glover (Univ. of Cambridge, UK), respectively. Drosdel and Exelixis deficiency stocks, all other mutants and balancer chromosomes marked with Twist-Gal4, UAS-GFP were from the Bloomington *Drosophila* Stock Center. The genomic coordinates for the deficiencies are summarized on the Bloomington *Drosophila* Stock Center web page (http://flystocks.bio.indiana.edu). Baz::GFP and Arm::CFP were on the same recombinant X-chromosome as described previously [51]. WT was *yellow white.*

### Cuticle preparations and scoring

Embryos were collected for 24 h at 25°C and then removed from adults and allowed to develop for another 48 h. Unhatched embryos were washed and dechorionated with 50% bleach. Dechorionated embryos were mounted on slides with Hoyer's mountant:Lactic acid (1∶1), and baked at 60°C overnight. Embryos were viewed and scored by two people simultaneously using a dual view Olympus BX41 microscope with 4x and 10x objective lenses. For quantification of cuticle phenotypes, 100–200 cuticles were counted per experiment and the percentages from two experiments were averaged for the final distributions. For quantification of hatch rates, 300 unhatched embryos were tested per experiment and percentages from two experiments were averaged for the final hatch rate (unfertilized eggs were identified after cuticle preparations and were excluded from the analysis).

### Embryo staining and fluorescence microscopy

Embryos developed for 3–6 h at 25°C were dechorionated and then fixed in 1∶1 3.7% formaldehyde in PBS:heptane for 20 minutes, and were devitellinized using methanol. Blocking and staining was performed with PBS/1% goat serum/0.1% Triton X-100. Embryos were stained with rat anti-DE-cadherin (DE-cad) antibodies (DCad2 at 1∶100; [52]), mouse anti-phosphohistone H3 antibodies (1∶1000; Cell Signaling) and Alexa546 and 647 secondary antibodies (Invitrogen). Embryos were mounted in Aqua Polymount (Polysciences, Inc.). Epifluorescence imaging was performed using an Olympus BX51 microscope at room temperature with a 60x oil objective. Images were captured using Evolution UF cooled monochrome camera and QCapture Pro software. Adobe Photoshop was used for contrast and brightness adjustments.

### Generating and expressing GFP fusion proteins

cDNAs were from the Canadian *Drosophila* Microarray Centre and the Drosophila Genomic Resource Center. They were amplified by PCR, cloned into gateway entry vectors, sequenced and recombined into gateway destination vectors to add C-terminal GFP and an upstream UAS sequence. The gateway destination vector was pPWG with the attB sequence inserted into its NsiI restriction site. Transgenic flies were generated by Genetic Services with transgenes inserted into the attp2 site. Transgenic flies were crossed to actin5C-Gal4 females (Bloomington *Drosophila* Stock Center) for imaging F1 embryos.

### Live imaging

Dechorionated embryos were mounted in halocarbon oil (series 700; Halocarbon Products) on petriPERM dishes (Sigma). Images were collected with a Quorum spinning disk confocal system (Quorum Technologies), at RT, with a Zeiss 63x (Plan-Apochromat; NA 1.4) objective, a piezo top plate, a Hamamatsu EM CCD camera and Volocity software (Improvision). Z-stacks were collected with 300 nm step sizes. In all experiments, the autofluorescent vitelline membrane of the egg shell was used as a marker for the apical surface of the cells found just below it.

## Results

### Known epithelial regulators enhance the *baz* cuticle phenotype

**Figure 1:**
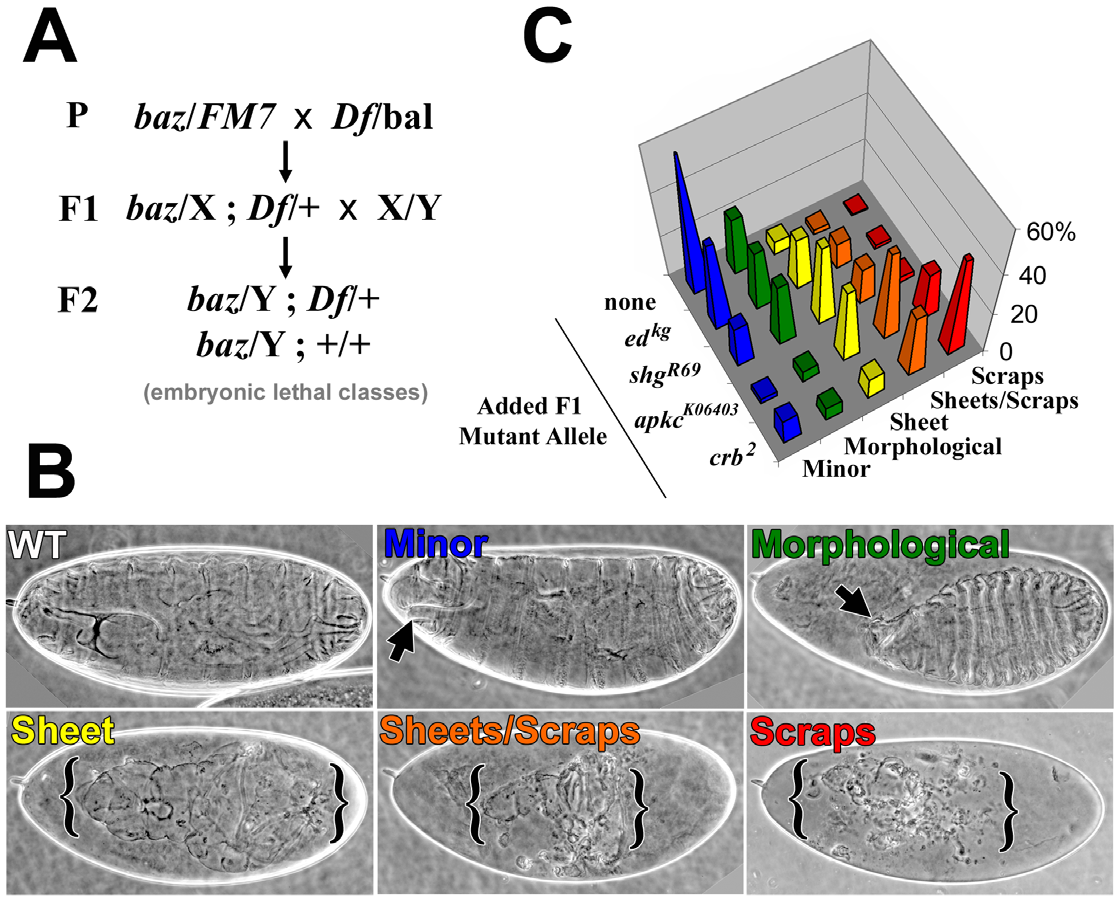
Examples of enhanced *baz* zygotic mutant cuticle phenotypes and a pilot screen. (A) Mating scheme to probe for dominant enhancement of the *baz* mutant phenotype. Abbreviations: Df (deficiency), bal (balancer chromosome). (B) Examples of cuticle phenotypes observed. A WT cuticle is shown. Disrupted cuticles were categorized from minor effects to scraps. Defects in minor and morphological categories indicated with arrows. Cuticle remaining in sheets, sheets and scarps, and scraps categories bracketed. Non-linear levels adjustments were done to accentuate the cuticle phenotypes without interference from the surrounding vitelline membrane (gamma values were set to 2.0 in Photoshop). (C) Cuticle phenotype distributions resulting from crossing mutant alleles for known epithelial regulators to *baz* mutants using the scheme in (A). The results are color-coded according to the classes shown in (B). The distribution of the control non-modified *baz* phenotypes is labeled ‘none’ because no additional mutant allele was added in the cross shown in (A). For these controls, the *baz* allele was out-crossed, and then the progeny of *baz*/X females and wildtype males were analyzed.

To assess whether a *baz* mutant enhancer screen would identify regulators of epithelial structure, we analyzed the effects of loss of function of four candidate genes on the *baz* zygotic mutant phenotype in a pilot screen. Specifically, we generated double heterozygote females for *baz* and each candidate gene, crossed them to WT males and analyzed the cuticle phenotypes of embryos that failed to hatch. In this scheme only hemizygous *baz* embryos (one quarter of the progeny) are expected to die (*baz* is X-linked). For all of these hemizygous *baz* embryos, maternal candidate interacting gene dosage is reduced by half, and for half of them, the zygotic candidate interacting gene dosage is also reduced by half (Fig. 1A).

First, we isogenized a stock carrying the strong hypomorphic *baz^Xi106^* allele (referred to as *baz* mutants), and analyzed the progeny of outcrossed *baz* mutant females crossed to WT males as a control. Dead embryos had largely intact cuticles with one, or two, small holes often at the head (Fig. 1B, C). This is likely due to defects in head morphogenesis rather than general break down of epithelial structure. We noticed that the presence of an X-chromosomal balancer enhanced the *baz* mutant phenotype (data not shown), possibly explaining why other studies reported a stronger zygotic phenotype for this allele [25].

Candidate gene mutations enhanced the *baz* mutant phenotype to varying degrees. We used the following categorization to classify defects (Fig. 1B): minor (outer cuticle largely intact with subtle head defects and/or missing head skeleton, arrow), morphological (head and/or dorsal holes due to failed epithelial rearrangements, arrow), sheet (a large sheet of cuticle likely resulting from major morphogenesis defects and some breakdown of basic epithelial structure, bracketed), sheets and scraps (substantial breakdown of basic epithelial structure, bracketed), scraps (major breakdown of basic epithelial structure resulting in only small residual epithelial structures capable of secreting cuticle, bracketed). The *crumbs* allele *crb^2^* produced the greatest enhancement of the *baz* mutant phenotype, followed by *apkc^K06403^*, the *shotgun* allele *shg^R69^* (*shg* encodes DE-cadherin, DE-cad) and the *echinoid* allele *ed^kg^* (Fig. 1C). Since reduced levels of *crb* and *apkc*, produced the scraps phenotype (Fig. 1B) in 44 and 21% of the dead progeny, respectively, we established the presence of >15–20% of dead embryos with the scraps phenotype as a threshold for selecting *baz* interactions. With this criterion, false negatives will occur, as *shg* and *ed* alleles only moderately enhanced the *baz* mutant phenotype. Also, our mating scheme prevented analysis of other X-linked genes (e.g. the epithelial regulators *par-6*, *cdc42*, *stardust* and *arm* could not be tested in our mating scheme because they are X-linked).

### Screening chromosome 2 and 3 deficiencies for enhancers of *baz*

**Figure 2:**
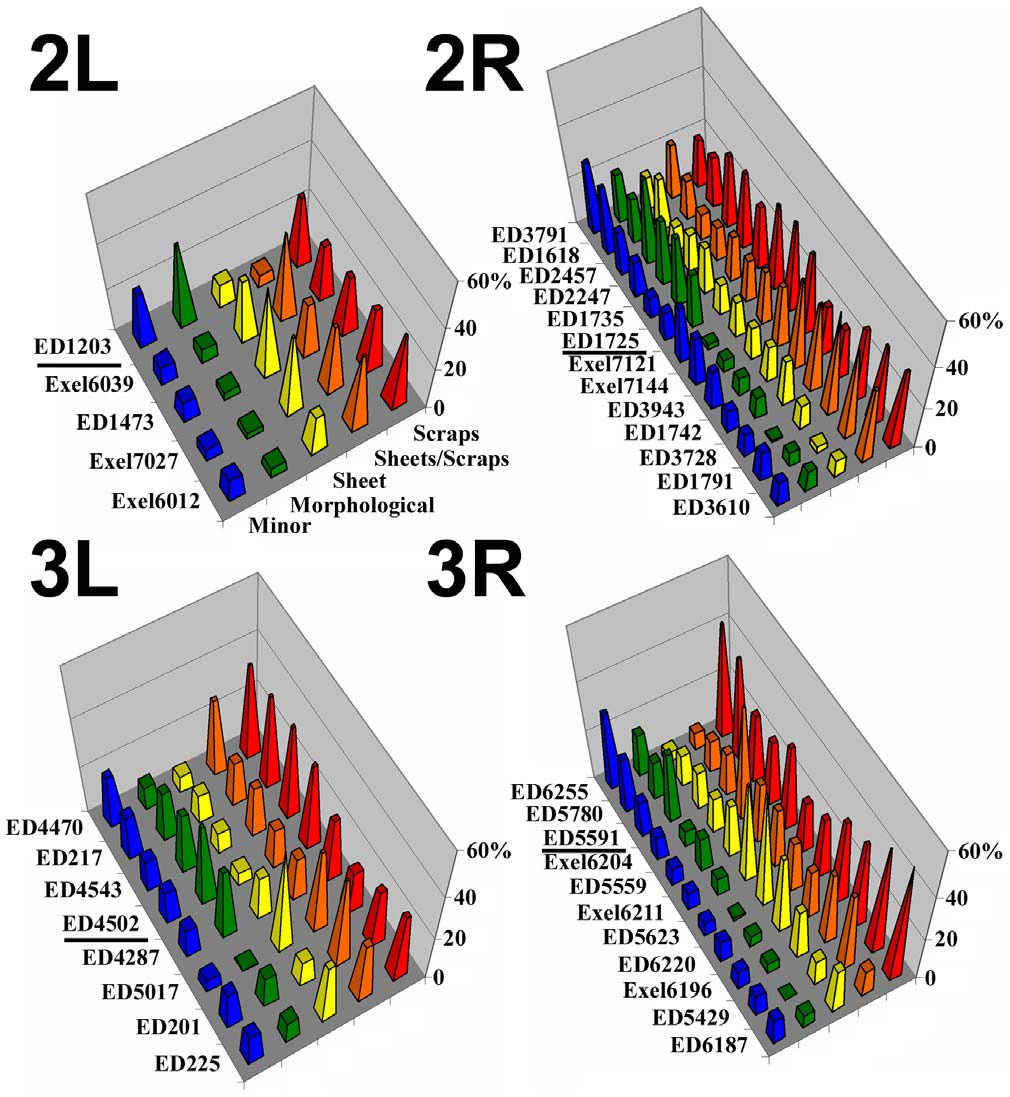
Distributions of *baz* zygotic mutant cuticle phenotypes enhanced by chromosome 2 and 3 deficiencies. Results are color-coded according to the classes shown in Fig. 1B. Each graph is organized with the strongest enhancements at the front (red peaks) and progressively weaker enhancements further back. A black horizontal bar separates interactions with more bimodal distributions to the back.

To screen for additional *baz*-interacting genes, we assembled a deficiency kit composed of molecularly defined Drosdel and Exelixis deficiencies available from the Bloomington *Drosophila* Stock Center. Since Drosdel deficiencies are generally larger, we first selected Drosdel deficiencies that gave the maximum coverage of chromosomes 2 and 3 with the minimal number of deficiencies. We then filled gaps where Exelixis deficiencies were available. Overall, this kit allowed us to screen 8514 of the 11466 protein coding genes on chromosomes 2 and 3 (74%) (Table 1). We began by screening dead F2 embryos (100–300 per cross) for an enhanced cuticle phenotype class making up at least 15–20% of the population. Of the 278 deficiencies screened, 26 had minimal effect, 93 produced enhanced morphogenesis defects in the most severe class, 122 produced embryos with detached sheets in the most severe class, and 37 produced embryos with small cuticle scraps in the most severe class (Table 1). Quantification of the strongest 37 interactions revealed a range of severity (Fig. 2). In some, the phenotypes of the majority of *baz* mutant embryos were enhanced to scraps or a mix of sheets and scraps. Others displayed a more even distribution across the phenotype spectrum or bimodal distributions, suggestive of two classes of interactions. The proportion of embryos displaying only scraps ranged from 17.2% to 51%, meeting our criterion for strong interactions based on our pilot screen.

**Table 1:** Distribution of *baz* mutant cuticle phenotype modifications by deficiencies.

	Protein Coding Genes	Protein Coding Genes Screened	Minor defects	Morphological	Sheets	Scraps
2L	2617	1812 (69%)	14	28	33	**5**
2R	2740	1936 (71%)	5	21	17	**13**
3L	2706	1981 (73%)	5	15	35	**8**
3R	3403	2785 (82%)	2	29	37	**11**
Total	11466	8514 (74%)	26	93	122	**37**

To test if the interactions were due to a combination of maternal and zygotic interactions or solely zygotic interactions we crossed *baz* heterozygous females to males heterozygous for each parent deficiency. This removes any maternal effect of the deficiencies. Only two of the deficiencies enhanced the *baz* phenotype to produce >15–20% dead embryos with the scraps phenotype, suggesting that most of the interactions involve maternal effects and thus may involve the earlier establishment of epithelial structure during embryogenesis (Table S1, column 4). Of note, the parent deficiency Exel6039 had non-maternal interaction with *baz* and contained the gene for muscle Myosin heavy chain. Reducing levels of muscle Myosin heavy chain also had a non-maternal interaction with *baz* producing dead embryos with the scraps phenotype (data not shown), suggesting a possible late interaction between muscle tissue and the epidermis. However, we focused on pursuing earlier interactions.

### Mapping and categorizing potential interacting genes

To identify the genes responsible for the enhanced *baz* mutant phenotypes, we first selected other Exelixis or Drosdel deficiencies that overlap with the 37 interacting deficiencies identified above to narrow the chromosomal interval (Table S1, columns 1–3). For 12 of the parent interacting deficiencies, overlapping deficiencies were not available. For four, the strong interaction was lost when multiple deficiencies that fully spanned the parent were analyzed, suggesting the original interaction was with two genes in the parent deficiency. For 18, a partially overlapping deficiency enhanced the *baz* mutant phenotype, allowing us to map the position of the interacting gene to a smaller chromosomal interval. For three, overlapping deficiencies did not enhance the *baz* mutant phenotype but deficiencies were unavailable to test all genes in the parent deficiency. For these three, we negatively mapped a shorter gene span that excluded genes in the non-interacting mapping deficiencies, but it is possible that the interaction involves more than one gene and might be lost if full coverage of the parent deficiency was possible.

We used bioinformatics to characterize gene function in the minimal mapped intervals. We first used Flybase to compile gene ontogeny data for molecular function, biological process and/or cellular component. If gene ontogeny data were not available, we performed a BLAST search to assign a possible function. If a possible function was still unclear, we used the BLAST search results to classify the gene as either unique to *Drosophila* (and closely related species (as far as mosquito)) or unknown but conserved among a range of species. For genes with functional information, we selected more relevant candidates (polarity, cytoskeletal, transmembrane, trafficking (involving possible plasma membrane interactions), and signaling (involving potential cytoskeletal effects)) and disregarded other categories (genes with unknown function, nucleic acid interacting genes, metabolic genes etc.) (Table S1, column 5). In total, we selected 86 more relevant candidates and disregarded 569 apparently less relevant candidates.

Since most of the interactions we discovered involved a maternal effect, we further reduced the list of 86 functionally relevant candidates by selecting those with significant mRNA expression in syncytial early embryos detected in either the expression studies of the Berkeley Drosophila Genome Center (genes with scores below 400 excluded) or the study by Pilot et al. [53] (low percentile ranks excluded) (Table S1, column 6). The results from each study were generally comparable, but in cases where one suggested early expression and the other did not the gene was considered expressed for our analysis. There was evidence for early embryo expression of 64 of the 86 functionally relevant candidates and these were pursued (Table S1, column 7).

### Identifying individual gene mutations that enhance the *baz* mutant phenotype

**Figure 3:**
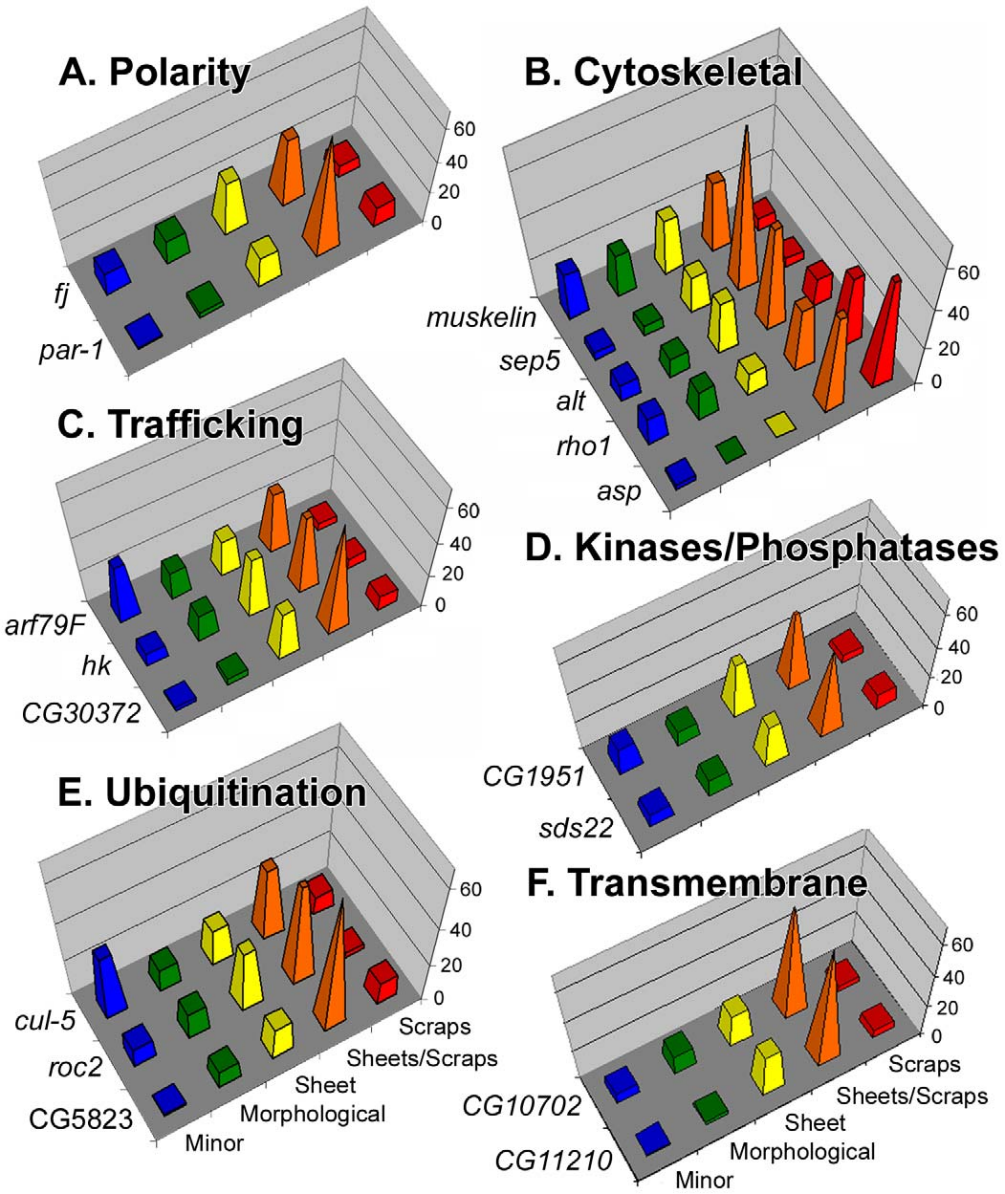
Cuticle phenotype distributions of *baz* zygotic mutants enhanced by individual gene mutants. Results are color-coded according to the classes shown in Fig. 1B. The individual genes are grouped into functional categories from Table S1. Each graph is organized with the strongest enhancements at the front (red peaks) and progressively weaker enhancements further back. Gene abbreviations used: *abnormal spindle* (*asp*), *aluminum tubes* (*alt*), *cullin-5* (*cul-5*), *four jointed* (*fj*), *hook* (*hk*), *septin 5* (*sep5*).

To identify genes within the deficiencies that interact with *baz*, we analyzed potential or known mutant alleles available from the Bloomington *Drosophila* Stock Center (Table S1, columns 8–9). For 23 of the 64 properly expressed and functionally relevant candidates, there were no stocks available. For 24, the available alleles produced no scraps when combined with *baz* in our mating scheme—they displayed no or more subtle interactions. For 17, the available alleles enhanced the *baz* phenotype producing a substantial proportion of embryos with sheets and scraps of cuticle (Fig. 3). However, only five of these 17 displayed >10% of dead embryos in the scraps class; the microtubule regulator *abnormal spindle* (*asp*) (51%), *rho1* (32.7%), the possible cytoskeletal protein *aluminum tubes* (*alt*) (13%), *par-1* (11.6%), and the possible ubiquitin conjugating enzyme *CG5823* (10.8%). Since null alleles were available for only two of the 41 mutants tested, the lack of strong phenotypes for many of the tested genotypes could be due to incomplete loss of function (Table S1, column 9). However, in three cases, multiple interacting genes were found for one deficiency (two deficiencies contained two interacting genes each, and one deficiency contained three interacting genes), suggesting that the stronger phenotype of the deficiency may arise in part from the reduction of multiple interacting genes. Each of the 17 mutants also enhanced the cuticle defects of a separate *baz* allele (*baz* ^GO484^) (data not shown).

### The identified genes interact with *baz* in regulating AJs in specific tissues

Maternal/zygotic *baz* mutants fail to properly position AJs prior to gastrulation resulting in severe disruption of epithelial structure and morphogenesis [19], [24], [54]. We wondered whether loss of interacting gene function in zygotic *baz* mutants might limit the ability of maternally supplied Baz to regulate AJs and whether this occurs during specific epithelial morphogenesis events. The *Drosophila* embryo body plan takes shape at gastrulation [55]. The ventral furrow internalizes the mesoderm, and the posterior midgut invaginates from the posterior pole. The lateral germband undergoes convergent extension along the anterior-posterior axis (germband extension), and simultaneously, flat and elongated amnioserosa cells form on the dorsal surface and fill the space between the ventral and dorsal halves of the fully extended germband. The germband ectoderm and regions of the head epidermis form the larval epidermis which produces the outer cuticle, but first they develop mitotic domains, undergo neuroblast delamination and rearrange during dorsal closure and head involution.

**Figure 4:**
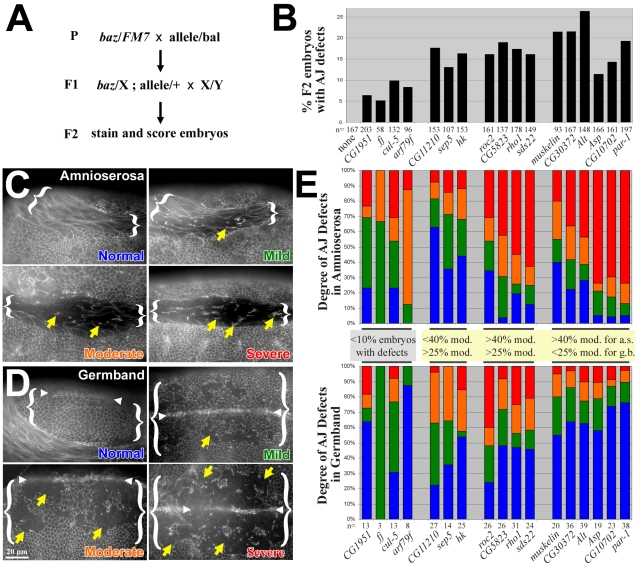
Assessing early AJ phenotypes of enhanced *baz* zygotic mutants. (A) Experimental set-up to analyze the DE-cad distribution of enhanced *baz* zygotic mutants in the F2 generation. (B) The percentage of F2 embryos displaying AJ defects detected by DE-cad staining. (C) The different types of amnioserosa AJ defects observed with DE-cad staining. The amnioserosa is bracketed. Lateral views of the embryonic body region are shown (dorsal is up and anterior is left). Normal, mild, moderate and severe defects are color-coded. Yellow arrow show abnormal clumps of DE-cad staining. DE-cad staining is lost from cell contacts in the moderate and severe categories. (D) The different types of germband AJ defects observed with DE-cad staining. The ventral neurectoderm region of the germband is bracketed. Dorsal views are shown with the anterior to the left (the germband is extended over the dorsal surface of the embryo at this stage). Normal, mild, moderate and severe defects are color-coded. Yellow arrows show groups of ventral cells that have lost DE-cad staining at cell contacts. DE-cad staining is lost from cell contacts of larger groups of cells in the moderate and severe categories. White arrowheads mark the ventral midline in each. The normal example is a lateral view showing one half of the epidermis from its most ventral edge (at the ventral mid-line) to its most dorsal edge (at the amnioserosa). The mild, moderate and severe examples focus on the ventral epidermis where the defects were seen. (E) The distribution of amnioserosa (a.s.) and germband (g.b.) AJ defects for each *baz* mutant enhancement identified. 4 groups are distinguished: (1) crosses which produced AJ defects in <10% of the F2 embryos (see panel B), (2) crosses which produced AJ defects in >10% of the F2 embryos (see panel B) in which <40% of amnioserosa defects were moderate or severe and >25% of germband defects were moderate or severe, (3) crosses which produced AJ defects in >10% of the F2 embryos (see panel B) in which >40% of amnioserosa defects were moderate or severe and >25% of germband defects were moderate or severe, and (4) crosses which produced AJ defects in >10% of the F2 embryos (see panel B) in which >40% of amnioserosa defects were moderate or severe and <25% of germband defects were moderate or severe. In each group the results are arranged in order of severity in the amnioserosa.

To specify when and how loss of interacting gene function enhances the *baz* mutant epithelial phenotype, we collected the F2 generation from our mating scheme 3–6 hours after egg laying and probed for AJ positioning using DE-cad immuno-fluorescence (Fig. 4A). We focused on stage 9–11 embryos in which the germband is extended and contains mitotic domains and delaminating neuroblasts, and in which the amnioserosa is fully formed. *baz* mutants alone displayed minimal defects at these stages (AJ defects were observed in 0/167 embryos of which 25% were *baz* zygotic mutants). Reducing the dosage of interacting genes in *baz* zygotic mutants did not block germband extension, suggesting that early morphogenesis was relatively normal. However, we found AJ fragmentation in stage 9–11 embryos. Quantifying all stage 9–11 embryos from these crosses, revealed a range of 5.3–26.4% with AJ fragmentation (Fig. 4B, 100–200 embryos from two separate experiments were scored for each cross). This was within the expected Mendelian ratio (25% are expected to be *baz* zygotic mutants with the dosage of an interacting gene decreased by ≤50%, see Fig 4A). A balancer chromosome was not used to identify the *baz* zygotic mutants because we found that it modifies both the *baz* mutant cuticle phenotype and the AJ phenotype on its own—this would thus confound the analyses of the *baz* interacting genes.

Next, we evaluated whether specific epithelia were affected in the enhanced *baz* phenotype. Minimal to severe AJ defects were observed in both the amnioserosa (Fig. 4C, tissue bracketed, arrows show abnormal AJ clustering) and the germband (Fig. 4D, tissue bracketed, arrows show cells missing AJs). In the germband, groups of cells had no AJs between them, suggestive of either mitotic domains or regions of neuroblast delamination (Fig. 4D, arrows). Double staining for DE-cad and phospho-histone H3 (a marker for mitotic chromosomes) showed that these cells were not mitotic (data not shown) suggesting the AJ defects may be associated with neuroblast delamination. This is consistent with past observations of AJ breakdown associated with neuroblast delamination after reducing DE-cad levels [56], although other explanations are possible.

The effects of the different genetic interactions were separated into groups based on the severity of overall AJ defects and tissue specific effects. For crosses which produced AJ defects in <10% of the F2 embryos, the amnioserosa and germband AJ defects were relatively non-severe (for *CG1951*, *four-jointed* (*fj*), *cullin-5* (*cul-5*) and *arf79f* mutant alleles) (Fig. 4E). For crosses which produced AJ defects in >10% of the F2 embryos we separated the interactions based on the relative effects on the amnioserosa and germband (Fig. 4E). Enhancement of the *baz* zygotic phenotype by *CG11210*, *septin 5* (*sep5*) and *hook* (*hk*) mutant alleles produced a relatively strong effect in the germband (<40% of amnioserosa defects were moderate or severe and >25% of germband defects were moderate or severe). Enhancement by *roc2*, *CG5823*, *rho1* and *sds22* mutant alleles had a relatively strong effect on both tissues (>40% of amnioserosa defects were moderate or severe and >25% of germband defects were moderate or severe). Enhancement by *muskelin*, *CG30372*, *alt*, *asp*, *CG10702* and *par-1* mutant alleles had a relatively strong effect on the amnioserosa (>40% of amnioserosa defects were moderate or severe and <25% of germband defects were moderate or severe). Thus, the identified genes interact directly or indirectly with *baz* to regulate proper AJ positioning. Additionally, they can be divided into groups that have differential affects on AJs in the amnioserosa and the neurectoderm in the *baz* mutant background.

### Cuticle phenotypes of single mutants for the *baz*-interacting genes

**Figure 5:**
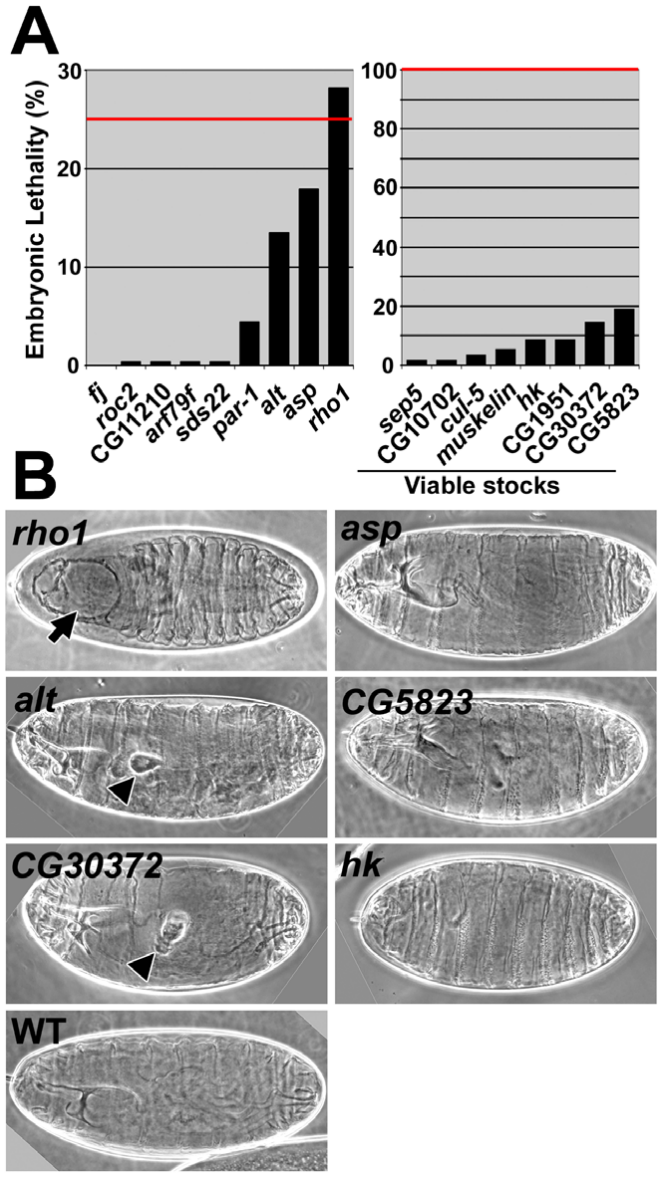
Cuticle phenotypes of individual zygotic mutants of *baz*-interacting genes. (A) Embryonic lethality rates. Left, non-viable mutants are shown. Alleles were out-crossed from balancer chromosomes before analysis. 25% percent of the population is expected to be homozygous mutant (indicated with red line). Right, viable mutants are shown. 100% of the population is homozygous mutant (indicated with red line). 300 embryos analyzed for each. (B) Cuticle phenotypes for mutants displaying >7.5% embryonic lethality.

To assess the phenotypes of each interacting gene individually, we began by assessing the terminal embryonic phenotypes of zygotic mutants. Nine of the mutant lines were homozygous lethal and three of these had >7.5% embryonic lethality (25% expected for full embryonic lethality; Fig. 5B). Eight of the mutant lines were viable and fertile as homozygotes, although four of them showed >7.5% embryonic lethality (100% were homozygous mutant embryos; Fig. 5A). Of the seven mutant lines with >7.5% embryonic lethality, *rho1* mutants displayed mainly holes in the head cuticle (Fig. 5B, arrows). *alt* and *CG30372* mutants had internal bulges apparently in the tracheal system (Fig. 5B, arrowheads). *asp*, *hk* and *CG5823* mutants had no apparent cuticle defects (Fig. 5B). *CG1951* mutants displayed a twisted sheet of cuticle, but this phenotype was not evident when the allele was placed in *trans* with a deficiency deleting CG1951 (data not shown). Thus, only *rho1*, *alt* and *CG30372* single mutants displayed epithelial phenotypes as terminal cuticle defects. These mild phenotypes may be due to hypomorphic alleles and/or the presence of maternal supplies of normal gene product.

To test if the single mutants had defects in Baz or AJ localization, we generated stocks for each mutant allele in which Baz::GFP and Arm::CFP were co-expressed at endogenous levels. All mutants were imaged live by 3-D live spinning disk confocal microscopy in the epidermis and amnioserosa at dorsal closure, but only *asp,* CG30372, *fj, hk, par-1, roc2,* and *sep5* displayed reduced levels of cortical Baz::GFP and Arm::CFP versus wild-type embryos co-expressing Baz::GFP and Arm::CFP (data not shown). To determine if endogenous Baz and Arm are also affected in the seven mutants, we immuno-stained mutant stocks without Baz::GFP and Arm::CFP. At dorsal closure, the cortical levels of endogenous Baz and Arm in the mutants were indistinguishable from WT (data not shown). This difference may be due to Baz::GFP and Arm::CFP being more sensitive to perturbation than their endogenous, untagged counterparts. However, it appears that stronger alleles will be needed to assess affects of these genes on endogenous Baz and AJ localization.

### Seven of the 17 interacting genes encode proteins that localize to the apical cortex

Immuno-staining studies have shown apical localization of both Rho-1 [57], [58] and Par-1 [59] in the *Drosophila* embryo. To determine the sub-cellular location of the other 15 genes, we generated GFP-tagged versions of the proteins, expressed them during embryogenesis, and performed live imaging of the epidermis at stage 15 (dorsal closure).

**Figure 6:**
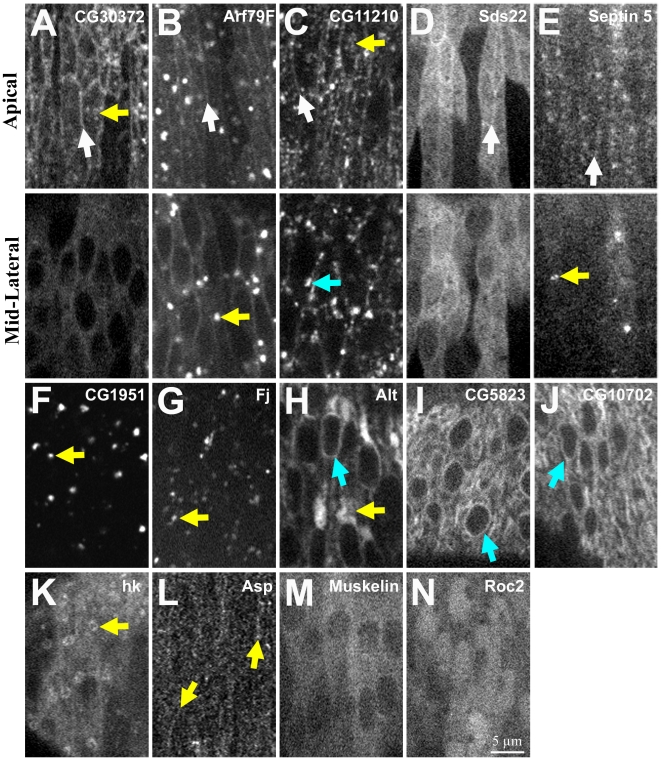
Localization of the proteins encoded by the *baz*-interacting genes. Live images of GFP-tagged versions of the proteins in lateral epidermal cells at stage 15 are shown. (A-E) Both apical sections and sections midway down the same cells are shown. (F-K and M-N) Sections midway down the cells are shown. (L) An apical section is shown. (A) CG30372::GFP around the apical circumference (white arrow) and at apical surface (yellow arrow). (B) Arf79F::GFP around apical circumference (white arrow) and at cytoplasmic puncta (yellow arrow). (C) CG11210::GFP around apical circumference (white arrow), at apical surface (yellow arrow) and at cytoplasmic puncta (cyan arrow). (D) Sds22::GFP around apical circumference (white arrow). (E) Septin 5::GFP around apical circumference (white arrow) and at cytoplasmic puncta (yellow arrow). (F-G) CG1951::GFP and Fj::GFP at cytoplasmic puncta (yellow arrows). (H-J) Alt::GFP, CG5823::GFP and CG10702::GFP over large cytoplasmic compartments (yellow arrow in H) and at nuclear membrane (cyan arrows). (K) hk::GFP at intermediate-sized compartments (yellow arrow). (L) Asp::GFP in parallel linear structures (yellow arrows). (M) Muskelin::GFP diffuse in cytoplasm. (N) Roc2::GFP diffusely in the cytoplasm and with nuclear enrichment.

Five additional proteins localized to the apical circumference. CG30372::GFP showed strong enrichment at the apical circumference (Fig. 6A, white arrow) and apical surface (Fig. 6A, yellow arrow) versus the basolateral cortex and the cytoplasm. Arf79F::GFP was also enriched at the apical circumference (Fig. 6B, white arrow) but not as strongly as CG30372::GFP. Arf79F::GFP also localized to punctate cytoplamic complexes/compartments (Fig. 6B, yellow arrow). CG11210::GFP was enriched at the apical circumference (Fig. 6C, white arrow) and to the apical surface (Fig. 6C, yellow arrow). CG11210::GFP also localized to punctate cytoplamic complexes/compartments (Fig. 6C, cyan arrow) with some similarities to those seen with Arf79F::GFP. Sds22::GFP displayed enrichment at the apical circumference (Fig. 6D, white arrow), a diffuse cytoplasmic distribution and some nuclear localization. Sep5::GFP had a punctate apical distribution that appeared to be around the cell circumference (Fig. 6E, white arrow) and also localized to cytoplasmic complexes/compartments (Fig. 6E, yellow arrow). Thus, CG30372, Arf79F, CG11210, Sds22 and Sep5 can be recruited to the apical domain of epithelial cells.

The remaining 10 proteins localized to various non-cortical compartments. CG1951::GFP and Fj::GFP labeled punctate cytoplasmic complexes/compartments (Fig. 6F-G, yellow arrows) similar to those labeled with Arf79F::GFP and CG11210::GFP. Alt::GFP, CG5823::GFP and CG10702::GFP localized to large cytoplasmic compartments that may be part of the ER (Fig. 6H-J, yellow arrow) and to the nuclear membrane (Fig. 6H-J, cyan arrows). hk::GFP localized to intermediate-sized compartments with hk::GFP-negative centers (Fig. 6K, yellow arrow). Asp::GFP was weakly detected but appeared to localize in parallel lines along the dorsal-ventral axis of the embryo after deconvolution (Fig. 6L, yellow arrows). Muskelin::GFP localized diffusely in the cytoplasm with nuclear exclusion (Fig. 6M). Roc2::GFP localized diffusely in the cytoplasm with nuclear enrichment and apparent nucleolar exclusion (Fig. 6N). Cul-5::GFP was undetectable. These proteins may affect epithelial structure through intracellular trafficking or signaling.

## Discussion

We identified 17 genes that interact with Baz to regulate epithelial structure. For 13 of these, this is the first report of a role in epithelial polarity. Use of a genetic modifier screen was key for implicating these genes as epithelial regulators, since single mutants for these genes had very subtle cuticle phenotypes with alleles available. Further implicating a role in epithelial polarity, seven of the 17 genes encode proteins that can be recruited to the apical domain of epithelial cells in the *Drosophila* embryo (Fig. 6; [57]–[59]).

Microarray data indicate that all 17 genes are expressed in early *Drosophila* embryos (Berkeley Drosophila Genome Center; [53]). mRNA localization data from the Berkeley Drosophila Genome Project and Flyfish [60] show that some of them are expressed in distinctive patterns. During cellularization and early gastrulation, *CG30372* is expressed as a wide central band ending at the anterior and posterior termini of the embryo, while *fj* is expressed in two stripes that appear to overlap the two ends of the *CG30372* band—these are interesting patterns given the role of the anterior-posterior patterning system in controlling Baz planar polarization and cell intercalation at gastrulation [45], [61]. *asp* mRNA is apical in both epithelial cells and in neuroblasts, similar to Baz mRNA and protein localization [62]. *CG5823*, *rho1*, *par-1*, *sds22* and *hk* mRNAs have ubiquitous expression at cellularization. *CG1951*, *CG11210* and *roc2* mRNAs are also in all cells at cellularization but are excluded from the apical domain. mRNA localization data was not available for the six other interacting genes (*cul-5*, *arf79f*, *sep5*, *muskelin*, *alt* and *CG10702*).

The genetic interactions identified in our screen appear to be especially important for regulating dynamic epithelia. We observed AJ disruption in both the amnioserosa and the neurectoderm. These tissues have specific demands for AJ remodeling. During gastrulation, the amnioserosa undergoes a transition from a columnar epithelium into a flattened squamous epithelium. The flattening of these cells greatly enlarges their circumferences and Baz has been shown to regulate AJ remodeling as this occurs [51]. In the neurectoderm, a reduction of DE-cad leads to loss of AJs because of dynamic AJ remodeling associated with neuroblast delamination [56]. Recently, Cdc42, PAR-6, aPKC and Baz have been shown to indirectly stabilize neurectoderm AJs by controlling the trafficking of Crb [48]. The proteins we have implicated appear to directly or indirectly affect AJs during these processes as well. The partial tissue specificity we observed may reflect separable regulatory networks important for AJ positioning in each tissue. Based on our localization studies, many of the proteins could act directly in the apical domain while the others may impact apical polarity indirectly from various intracellular sites.

### Polarity proteins

In our pilot screen we found that reduction of *apkc* or *crb* substantially enhances the *baz* mutant cuticle phenotype. Our deficiency screen also found genetic interactions with *par-1* and *fj*. Baz/PAR-3 is known to interact with aPKC in a complex with PAR-6 to regulate cell polarity in many contexts [15]. In the follicular epithelium, PAR-1 has been shown to localize the basolateral membranes where it phosphorylates and inhibits Baz to maintain apical Baz polarity [27]. Similarly, knock-down of PAR-1 in the early embryo leads to abnormal spreading of AJs in the apicolateral region, but in embryonic epithelia PAR-1 is enriched in the apicolateral region versus the basolateral domain [59].

Although direct links between Baz and the planar polarity regulator *fj* have not been made, Baz localizes in a planar polarized pattern during germband extension [45]. Germband extension occurs independently of the canonical planar polarity genes Frizzled and Dishevelled [45], but to our knowledge, other planar cell polarity genes, such as *fj*, have not been tested. As discussed above, *fj* has an intriguing striped mRNA expression pattern at this stage, suggesting a link to the A-P patterning system, which regulates planar polarity in the tissue [45]. Fj is a golgi-associated protein, consistent with our localization data, which can phosphorylate transmembrane proteins en route to the plasma membrane [63]. Thus, Fj may affect the apical domain via transport from the Golgi.

### Intracellular trafficking proteins

Intracellular membrane trafficking plays a central role in controlling epithelial cell polarity [8] and AJs [64]. More specifically, Baz/PAR-3 and its interaction partners PAR-6 and aPKC have been implicated in regulating the endocytosis of apical proteins and AJs in *Drosophila* epithelia [46]–[48] and to impact general endocytic traffic in C. elegans [65]. Thus, we were interested in pursuing genes implicated in trafficking by gene ontogeny. We also found a number of additional proteins that appear to localize to intracellular compartments. Three of these localized to the apical cortex as well.

We found that Arf79F and CG30372 can localize to the apical domain. Generally, Arfs function in the formation and targeting of vesicles in the cell [66]. Arf79F is the *Drosophila* version of Arf1 and has been implicated in lipid droplet transport [67] and the regulation of the apical domain during *Drosophila* rhabdomere formation [68]. Intriguingly, CG30372, encodes a putative ArfGAP. Although not characterized in *Drosophila*, CG30372 has a similar domain structure to the ASAP proteins (Arf GAPs with Src homology 3, ankyrin repeat, and pleckstrin homology domains), which have been implicated in the regulation of actin and endocytosis [69]. It will be interesting to test whether Arf79F and *Drosophila* ASAP interact to regulate epithelial structure. Of note, CG11210::GFP has a similar distribution as Arf79F, localizing to the apical cortex and intracellular compartments. CG11210 is an uncharacterized protein predicted to have 10–11 transmembrane helices. We hypothesize that these proteins may co-ordinate membrane trafficking with the apical cortex.

Five other proteins localized to intracellular compartments without apparent cortical localization. As discussed, Fj appears to localize to the Golgi. CG1951, an uncharacterized kinase, appears to localize to scattered small vesicles. Alt, CG5823 and CG10702 appear to localize to ER membranes. Alt is functionally uncharacterized, but displays some sequence similarity with Myosins and MT associated proteins CLP190 and NUMA (BLAST search) and has been co-fractionated with lipid droplets from early embryos [70]. CG5823 has been implicated in ubiquitination (Flybase annotation), and CG10702 is a predicted receptor tyrosine kinase (Flybase annotation). hk localizes to intermediate sized vesicles consistent with past localization studies in other *Drosophila* cell types and hk's role in trafficking to the multivesicular body [71]. These five proteins might affect cell polarity through intracellular trafficking.

### Cytoskeletal proteins

The cytoskeleton also plays a major role in regulating epithelial structure. Our screen found that *rho1*, *sep5* and *asp* genetically interact with *baz.* Rho1 localizes to the apical domain and other parts of *Drosophila* embryonic epithelia, and has been shown to have a general role in regulating epithelial structure [57], [58]. We also found that Sep5 can be recruited to the apical domain. In mammalian cells, Septin 2 has been shown to regulate AJs [72]. Asp functions at the centrosomes to control the structure of the mitotic spindle [73]. At stage 15, we detected Asp::GFP in linear parallel arrays consistent with the organization of MTs in these cells [74]. Thus, we speculate that Asp affects cell polarity via MTs. Muskelin is functionally uncharacterized, but contains kelch motifs found in cytoskeletal and other proteins [75]. However, Muskelin::GFP localized diffusely through the cytoplasm.

### Signaling proteins

We found that Sds22 localizes to the apical domain of embryonic epithelial cells. Sds22, a regulatory subunit of protein phosphatase 1 (PP1), has recently been linked to regulation of cell shape and apical-basal polarity in *Drosophila* imaginal disc and follicular epithelia, where a GFP-tagged form of Sds22 localized to the cytoplasm and nucleus [76]. Sds22 binds to all four Drosophila PP1 isoforms, and *sds22* phenotypes correlated with elevated phosphorylation of Myosin regulatory light chain and Moesin [76]. Intriguingly, PP1alpha has been shown to de-phosphorylate PAR-3 and affect tight junction formation in mammalian cell culture [77].

Cul-5, Roc2 and CG5823 are involved in protein ubiquitination. PAR-3 has been shown to interact with a ubiquitin ligase in the generation of neuronal polarity [41] and ubiquitination has also been linked to polarized cell protrusion [78]. Cul-5 regulates the neuromuscular junction in *Drosophila* [79], but roles for Cul-5, Roc2 and CG5823 in epithelial structure have not been described. Intriguingly, Roc2 and Cul-5 form a complex in *Drosophila* [80]. Perhaps this complex supports epithelial structure by down-regulating inhibitors of the apical domain.

In this screen, we sought to identify additional proteins that function with Baz to regulate epithelial structure in the *Drosophila* embryo. From the 655 possible interacting genes identified through our deletion screening and mapping, we used gene ontogeny terms to select genes with possible functions in polarity, the cytoskeleton, membrane trafficking or signaling, as well as transmembrane proteins. We did this based on the known roles for Baz/PAR-3 in controlling cell structure at the cortex, but our approach would miss novel Baz functions and interactions with genes with unknown function or that are unique to *Drosophila*. Nonetheless, 13 of the 17 genes implicated by our screen have not been previously shown to interact with Baz or to affect epithelial structure, and thus should be of interest for future studies.

## Supporting Information

Table S1 Genetic Mapping and Bioinformatic Analysis of Interacting Deficiencies.
(0.02 MB PDF)
