# Supplementary material for: A Modifier Screen for Bazooka/PAR-3 Interacting Genes in the Drosophila Embryo Epithelium
Source: PLoS One. 2010 Apr 1;5(4):e9938. doi: 10.1371/journal.pone.0009938 (PMC2848566; doi:10.1371/journal.pone.0009938)
Supplement: Table S1 — Genetic Mapping and Bioinformatic Analysis of Interacting Deficiencies. (0.02 MB PDF) [file pone.0009938.s001.pdf]

Table S1. Genetic Mapping and Bioinformatic Analysis of Interacting Deficiencies

Parent Deficiency	Mapping Results (1)	Mapped Gene Interval	Zygotic Interaction (Solely)	Unique to Drosophila	Unknown Function/Conserved	DNA/RNA/Ribosome Binding	Metabolism(2)	Organelles (3)	Proteasome	Signaling (4)	Larval/Adult Functions	ECM and Non-epidermal	Improperly Expressed Candidates (5)	Candidates	Basic Function (6)	Interaction	Allele (Null?)
**2L**																	
Exel6012	none	CG14029-CG6907		1	2	4	6	1	0	0	2	0	0	***CG7371***	Cytoskeleton	minimal	f02109 (unknown)
Exel7027	none	CG11181-CG11326		1	0	2	3	0	0	1	0	1	0				
ED1473	**LOST**																
Exel6039	Exel7067	CG31782-CG13279	**YES**	0	2	6	3	3	1	0	0	0	1				
ED1203	ED1200	CG15171-CG17344		3	2	2	7	0	1	1	1	0		***hk***	Trafficking	**YES**	1 (unknown)
														CG15173	Cytoskeleton	minimal	f05160 (unknown)
														robl37BC	Cytoskeleton	*no stock*	
														CG10702	Transmembrane	**YES**	f03893 (unknown)
														CG10470	Transmembrane	*no stock*	
														CG10493	Transmembrane	*no stock*	
**2R**																	
ED3610	ED3636	CG10917-CG5170		8	1	2	10	3	0	2	7	0	0	***fj***	**Polarity**	**YES**	d1 (unknown)
ED1791	none	CG8029-CG1888		11	1	3	9	3	0	5	1	0	3	***pkn***	Signaling	minimal	06736 (yes)
														brp	Cytoskeleton	*no stock*	
ED3728	none	CG8201-CG8517		10	5	2	3	0	0	2	6	0	1	***par-1***	**Polarity**	**YES**	w3 (unknown)
														CG11242	Cytoskeleton	minimal	e04210 (unknown)
														rep	Trafficking	minimal	f01510 (unknown)
ED1742	Exel7096	CG2060-CG2397		1	0	0	14	0	0	0	0	0	0	***mXr***	Transmembrane	minimal	DG17503 (unknown)
ED3943	Exel6077	CG17950-CG13493		3	2	3	7	2	0	0	0	0	0	***CG30398***	Cytoskeleton	*no stock*	
Exel7144	none	CG4927-CG5072		1	0	0	0	0	0	0	0	0	1				
Exel7121	none	CG8592-CG8772		2	0	5	8	0	0	0	0	0	2	***muskelin***	Cytoskeleton	**YES**	f04338 (unknown)
														Galpha49B	Signaling	minimal	f04219 (unknown)
														amph	Trafficking	minimal	26 (unknown)
ED1725	Exel6055	CG30377-CG12769		3	4	5	10	2		2	1	0	1	***sep5***	Cytoskeleton	**YES**	f04717 (unknown)
ED1735	Exel7094	CG17977-CG2158		3	1	0	4	2	1	0	1	0	0	***CG11210***	Transmembrane	**YES**	KG08546 (unknown)
														CG30372	Trafficking	**YES**	KG03963 (unknown)
ED2247	none	CG30035-CG8972		1	5	5	10	1	1	6	1	0	2	***CG13197***	Signaling	*no stock*	
														roc2	Signaling	**YES**	EP2487 (unknown)
														CG13192	Signaling	minimal	EY07746 (unknown)
ED2457	none	CG8370-CG30095		0	4	3	3	1	1	1	3	2		***rho1***	Cytoskeleton	**YES**	72O (unknown)
														CG8397	Cytoskeleton	*no stock*	
														ric	Trafficking	*no stock*	
ED1618	**LOST**																
ED3791	Exel6071	CG11312-CG3722		1	4	7	6	1	1	3	2	0	5	***shg***	Transmembrane	minimal	R69 (yes)
														sktl	Signaling	minimal	k12405 (unknown)
														lpk1	Signaling	*no stock*	
**3L**																	
ED225	none	CG13701-CG4306		0	2	0	0	0	0	1	0	0	0				
ED201	Exel6084	CG13875-CG7004		5	3	6	12	2	0	1	0	0	0	***CG7028***	Transmembrane	*no stock*	
														mthl14	Transmembrane	*no stock*	
														rhoGEF3	Signaling	*no stock*	
														fwd	Signaling	minimal	neo1 (unknown)
ED5017	ED231	CG8385-CG11100		1	0	6	1	0	0	0	0	0	0	***arf79F***	Trafficking	minimal	C005 (unknown)
ED4287	ED4284	CG12019-CG1828		4	3	3	1	0	0	1	3	0	0	***patj***	**Polarity**	*no stock*	
														R	Signaling	*no stock*	
ED4502	ED4515	CG32137-CG9040		3	2	2	1	0	0	0	0	0	1				
ED4543																	
ED4470	none	CG7628-CG6097		7	6	2	12	1	1	2	0	0	3	***srt***	Transmembrane	*no stock*	
ED217	**LOST**																
**3R**																	
ED6187	Exel8178	CG31128-CG13618	**YES**	2	0	1	0	0	0	0	3	0	0	***pp1alpha-96A***	Signaling	minimal	EY12810 (unknown)
ED5429	Exel6264	CG8327-CG31352		0	3	1	5	0	0	1	0	0	1	***eca***	Trafficking	minimal	EP469 (unknown)
														unc-115	Cytoskeleton	*no stock*	
														CG9492	Cytoskeleton	minimal	KG02504 (unknown)
														CG31352	Cytoskeleton	minimal	KG03651 (unknown)
														p24-2	Transmembrane	*no stock*	
Exel6196	none	CG5320-CG5405		1	3	2	2	1	1	0	0	0	0	***rab7***	Trafficking	minimal	EY10675 (unknown)
														CG31140	Signaling	*no stock*	
														AP-1sigma	Trafficking	*no stock*	
ED6220	negative	CG6677-CG13633		6	18	12	18	0	0	2	2	2	0	***asp***	Cytoskeleton	**YES**	1 (no)
		CG11069-CG11848												***CG10848***	Cytoskeleton	*no stock*	
														ssh	Cytoskeleton	minimal	1-63 (unknown)
														bai	Trafficking	*no stock*	
														veli	**Polarity**	*no stock*	
														CG31108	Cytoskeleton	minimal	KG01044 (unknown)
ED5623	**LOST**																
Exel6211	none	CG14519-CG1401		2	0	5	5	0	0	2	0	0	0	***pkc98E***	Signaling	*no stock*	
														cul-5	Signaling	**YES**	EY00051 (unknown)
														CG1951	Trafficking	**YES**	EY00129 (unknown)
ED5559	Exel7316	CG11466-CG5887		1	0	3	9	0	1	1	0	1	0	***pp1-87B***	Cytoskeleton	minimal	1 (no)
ED5591																	
Exel6204	negative	CG8337-CG8354		0	0	0	0	0	0	7	0	0	0				
ED5780	negative	CG11896-CG18212		10	11	4	13	0	1	5	4	0	0	***CG11896***	Cytoskeleton	minimal	d05816 (unknown)
														sds22	Signaling	**YES**	e00975 (unknown)
														CG5823	Signaling	**YES**	f04935 (unknown)
														CG31190	Transmembrane	minimal	C229 (unknown)
														alt	Cytoskeleton	**YES**	KG08013 (unknown)
ED6255	ED6235	CG5490-CG5521		3	2	6	2	1	0	1	2	1	1	***CG5521***	Signaling	minimal	KG08651 (unknown)
														tsp97E	Transmembrane	*no stock*	
		TOTALs		92	84	96	175	23	10	46	37	6	22	**64**			

(1) smaller interacting deficiencies indicated; none (no mapping possible with available deficiencies); LOST (non-interacting multiple deficiencies fully spanned the parent)
; negative (non-interacting deficiencies did not fully span the parent allowing negative mapping to other intervals)
(2) includes calcium binding and heat shock proteins
(3) membrane organelles except trafficking vesicles that interact with the plasma membrane
(4) signaling pathways to the nucleus or controlling the cell cycle or apoptosis
(5) early embryo mRNA expression studies of the Berkeley Drosophila Genome Center (scores below 400) or Pilot et al. (low percentile ranks)
(6) trafficking involving possible plasma membrane interactions and signaling involving potential cytoskeletal effects
